# Evolving beyond TB: launch of a new journal for chronic respiratory disease

**DOI:** 10.5588/ijtldopen.25.0779

**Published:** 2025-12-10

**Authors:** H.D. Blackbourn, G.B. Migliori

**Affiliations:** ^1^International Union Against Tuberculosis and Lung Disease, Paris, France;; ^2^Istituti Clinici Scientifici Maugeri, Istituto di Ricovero e Cura a Carattere Scientifico, Tradate, Italy.

**Keywords:** tuberculosis, IJTLD CRD, COPD, asthma, smoking, air quality, bronchiectasis, LMICs, open access

## Abstract

After the positive experience of launching *IJTLD Open*, we are now embarking on the launch of a new journal to focus on chronic respiratory diseases, *IJTLD CRD*. Chronic conditions have been relatively negelected and the journal will be a home for all open access articles on topics to include post-TB lung disease, COPD, asthma, impact of air pollution, lung damage in smokers, bronchiectasis, and occupational lung disease. By highlighting research on these different conditions, we hope to identify commonalities and speed up the development and adoption of suitable treatments. This will also allow the *International Journal of Tuberculosis and Lung Disease* (*IJTLD*) and *IJTLD Open* to focus on infectious disease, with expanded coverage of bacterial, viral and fungal respiratory diseases.

The *International Journal of Tuberculosis and Lung Disease* (*IJTLD*) was launched over a century ago as the flagship journal for the Union to communicate the latest advances in TB research. From the outset, the intention was for the Journal to serve the entire TB community and, as the fight to end TB has become a global effort, so its audience has grown. In part this is because the journal is freely available to Union members, but also because of access via institutional subscriptions. Fast forward 100 years and, in December 2023, we were on the cusp of launching *IJTLD Open*.^1^ Happily, this new journal has been well received, and surprised even us with its reach and impact.^2^ Unrestricted access allows anyone with an interest to read the latest research, and pivotal to this is the role that PubMed Central plays as a hub for readers from low- and middle-income countries (LMICs).^3^

The two journals primarily focus on TB in LMICs, and we are extremely grateful to our authors for submitting their manuscripts on all aspects of TB control. As awareness has grown of post-TB lung disease (PTLD) we have also published a broad range of articles on this topic,^4–9^ with articles on aspergillosis,^10^ asthma,^11,12^ COPD,^13,14^ air pollution,^15,16^ and sleep apnea.^17,18^ However, we are keenly aware that we have not promoted these chronic respiratory diseases (CRDs) as effectively as we have TB research. For example, globally, there are over 4 million deaths due to CRDs each year, with a prevalence of over 400 million cases;^19–21^ for COPD alone, this is predicted to rise to 600 million cases by 2050.^22^ Unfortunately, although the commercial publishers cover CRDs in high-income countries, the conditions in LMICs are relatively neglected, leaving a huge gap in our understanding. Given the Union’s mission to ‘end suffering due to lung diseases’ we can no longer ignore the impact of CRDs on many millions of people. We are therefore delighted to announce the launch of a new sister journal, *IJTLD Chronic Respiratory Disease* (*IJTLD CRD*). The journal will launch in 2026 as a quarterly open access journal, with the ability to grow as interest develops. Our aim is to make this journal a home for research on CRDs in LMICs. PTLD provides a natural bridge from our traditional coverage of TB, and each issue will also include articles on asthma, COPD, impact of air pollution, lung damage in smokers, bronchiectasis, and occupational lung disease. By highlighting this research, we will raise the profile of the different conditions, allowing researchers, clinicians, civil society, and policymakers to improve diagnosis and support for the millions of people who lack appropriate care. We also hope to identify commonalities and promote effective approaches for pulmonary rehabilitation.

Because traditional subscription journals are not available to readers in LMICs, the new journal is open access. However, we recognise that limited funding is available to researchers in these fields and have set article processing charges accordingly. Also, as we have done for *IJTLD Open*, we will continue to offer the *IJTLD* as a free-to-publish model for authors from LMICs who lack funding. To ensure the quality of content, *IJTLD CRD* will have the same values, scope and Editorial Board as its sister titles, with the same peer review process and acceptance criteria. Authors will submit their manuscripts via the same portal and manuscripts will be evaluated solely on scientific merit. After a paper has been accepted, authors with funding will be published in *IJTLD CRD*, whereas those without will be published in the IJTLD. We believe this will further improve access to important scientific knowledge.

The new journal also presents an opportunity for the *IJTLD* and *IJTLD Open* to focus on a broader range of infectious respiratory diseases. We had already begun this process, with articles on non-tuberculous mycobacteria (NTMs), aspergillosis and viral infections, including COVID-19.^23–26^ We will now engage with these topics more fully, with expanded coverage of all bacterial, viral and fungal respiratory diseases.

We remain indebted to our Editorial Board, peer reviewers, authors and readers in helping us to fulfil our mission – and thank all of you for your support!
